# Network analysis of core symptom changes in Chinese patients undergoing radical prostatectomy for prostate cancer: a longitudinal study

**DOI:** 10.3389/fonc.2026.1843406

**Published:** 2026-07-29

**Authors:** Xin Huang, Xiaoqin Huang, Yiqin Shi, Wang Li, Guihong Zheng

**Affiliations:** 1 Department of Urology, The Second Affiliated Hospital of Chongqing Medical University, Chongqing, China; 2 Acute and Critical Care Nursing Innovation and Translation Engineering Research Center of Chongqing Education Commission of China, Chongqing, China; 3 Chongqing Geriatric Specialized Nursing Clinical Diagnosis and Treatment Center, Chongqing, China

**Keywords:** core symptoms, longitudinal study, network analysis, radical prostatectomy, symptoms

## Abstract

**Objective:**

To construct symptom networks at different time points during the early postoperative period in patients undergoing radical prostatectomy and to identify the core symptom at each stage.

**Methods:**

This longitudinal study used convenience sampling to recruit patients who underwent radical prostatectomy for prostate cancer in the urology departments of two tertiary grade A hospitals in Chongqing, China, between August 2024 and September 2025. Data were collected at 7 days (T1), 1 month (T2), and 3 months (T3) after surgery using a general information questionnaire and the Chinese version of the Memorial Symptom Assessment Scale. Symptom networks were estimated using R, and strength centrality was used to identify the core symptom at each time point.

**Results:**

Symptom network analysis showed that the T1 network included 12 nodes, with pain having the highest strength centrality (rs = 1.76), identifying it as the core symptom at 7 days after surgery. The T2 network included 10 nodes, with worrying having the highest strength centrality (rs = 1.28), making it the core symptom at 1 month after surgery. The T3 network also included 10 nodes, and worrying remained the most central symptom (rs = 1.32), identifying it as the core symptom at 3 months after surgery.

**Conclusion:**

Core symptoms differed across stages of early postoperative recovery in radical prostatectomy patients. Healthcare professionals should develop continuous, anticipatory, and targeted intervention strategies based on the core symptoms identified at each time point, identify symptom risks as early as possible, and implement precision symptom management to improve patients’ symptom experience during early postoperative recovery and enhance the efficiency of symptom management.

## 1 Introduction

Prostate cancer (PC) is one of the most common malignancies of the genitourinary system in older men and has become a major public health concern affecting men’s health worldwide. Projections suggest that by 2040, the annual number of new prostate cancer cases globally will rise from approximately 1.4 million in 2020 to about 2.9 million (1). As public health awareness has increased and diagnostic techniques have advanced, more patients with prostate cancer are being identified at an earlier stage and are therefore more likely to receive surgical treatment. Radical prostatectomy (RP) is a guideline recommended standard treatment for localized prostate cancer and selected cases of locally advanced prostate cancer. In addition to achieving tumor control, it can preserve organ function to some extent and reduce the risks of local recurrence and distant metastasis, thereby improving overall patient prognosis (2, 3).

Although radical prostatectomy is effective for tumor control, patients often experience a wide range of symptoms during the perioperative period and postoperative recovery because of surgical trauma, damage to muscular tissue or neural structures, and the effects of adjuvant therapies such as radiotherapy and endocrine therapy after surgery. These symptoms include both general cancer related symptoms and prostate cancer specific symptoms (4–6). During the preoperative waiting period, patients may experience psychological symptoms such as tension, anxiety, and sadness because of concerns about the disease, limited understanding of treatment, and uncertainty about the future (7, 8). The period from surgery to hospital discharge is typically the time of greatest symptom burden. Having just undergone surgery, patients are often in a state of metabolic and physiological imbalance (9, 10), which makes them more susceptible to discomforts such as pain, abdominal distention, sleep disturbance, nausea and vomiting, and dizziness (11, 12).

As patients move into the transitional phase after discharge and before functional recovery is fully established, acute postoperative reactions gradually subside, but recovery remains incomplete and unstable. At this stage, prostate cancer specific problems such as urinary incontinence, irritative urinary symptoms, and sexual dysfunction often remain prominent. Among these, urinary incontinence and sexual dysfunction are among the most common functional outcomes after radical prostatectomy and are especially pronounced during the first 3 months after surgery (13–15). At the same time, some patients may still require adjuvant treatments such as radiotherapy (16) or endocrine therapy (17), and treatment related adverse effects may further compound fatigue, urinary irritation, and hormone related discomfort, thereby increasing the overall symptom burden and negatively affecting recovery and quality of life.These symptoms do not occur in isolation. Rather, they may coexist within the same period and interact with one another, jointly influencing patients’ self management, social functioning, and recovery trajectory. Previous research has shown that symptoms such as emotional distress and fatigue are complexly interrelated with symptoms including urinary incontinence and pain (18). Overall, the symptom experience of radical prostatectomy patients is not characterized by isolated complaints, but more often by the coexistence and interaction of multiple symptoms. The cumulative impact of these symptoms not only impedes postoperative recovery, but also undermines quality of life, psychosocial functioning, and self management capacity, thereby increasing the complexity of clinical symptom management.

At present, research on symptoms after radical prostatectomy, both in China and internationally, still focuses primarily on individual symptoms, with particular attention to urinary incontinence, sexual dysfunction, anxiety, and depression. Most studies have centered on symptom prevalence, influencing factors, and intervention effects, and these findings have provided an important basis for identifying key clinical problems and developing targeted interventions. However, existing studies have largely treated symptoms as independent outcome variables and have paid limited attention to the coexistence of multiple symptoms and their interactions within the same stage of recovery. As a result, they have not fully captured the complexity of patients’ real symptom experience during recovery. This limitation is especially evident during the period from before surgery to 3 months after surgery, when symptoms change rapidly and quality of life fluctuates substantially. Longitudinal studies examining symptom characteristics at different time points and shifts in core symptoms across stages remain relatively scarce.

Network analysis (NA), an important method introduced into symptom research in recent years, has provided a new framework for understanding the complex relationships among multiple symptoms (19, 20). Based on graph theory, this approach conceptualizes symptoms as nodes in a network and the associations between symptoms as edges. By estimating conditional dependence relationships, network analysis constructs a symptom network that more directly captures the underlying structure of intersymptom associations (21). Compared with traditional analytical approaches, network analysis not only visualizes the complex relationships among symptoms, but also identifies core symptoms that occupy key positions in the network through centrality measures. Core symptoms are typically strongly connected to multiple other symptoms and therefore exert greater influence on the overall symptom network and its changes over time. Wu et al. found that (22) chest pain occupied a central position in the symptom network of patients with heart failure, and that interventions targeting this core symptom could help reduce the severity of the overall symptom network while improving the specificity and effectiveness of symptom management. Accordingly, interventions centered on core symptoms may not only alleviate the target symptom itself, but also promote improvement in other related symptoms through network spillover effects, thereby enhancing the overall efficiency of symptom management.

Based on this, the present study aims to use network analysis to construct symptom networks at different time points during the early postoperative period in radical prostatectomy patients, compare changes in the structure of symptom interrelationships across stages, and identify the core symptoms at each stage. The findings are expected to provide both a theoretical basis and practical guidance for precision symptom management in radical prostatectomy patients during early postoperative recovery.

## 2 Participants and methods

### 2.1 Participants

This was a longitudinal study. Using convenience sampling, patients with prostate cancer who underwent surgical treatment in the departments of urology at two tertiary Grade A hospitals in Chongqing, China, between August 2024 and September 2025 were recruited as study participants. The inclusion criteria were as follows: (1) pathologically confirmed prostate cancer; (2) age 18 years or older; (3) undergoing radical prostatectomy for the first time; (4) ability to communicate and understand the study content; (5) provision of informed consent and voluntary participation in the study. The exclusion criteria were as follows: (1) severe complications or serious psychiatric disorders that precluded participation in the study; (2) coexisting malignant tumors or other life threatening diseases. This study was approved by the Ethics Committee of the Second Affiliated Hospital of Chongqing Medical University (Approval No. 2024-Yan Lunshen (89)).

The primary statistical method used in this study was network analysis. At present, there is no universally accepted standard for formal sample size calculation in network analysis. Epskamp et al. (23), pioneers in this field, have suggested that model stability should be a key consideration, with a minimum stability coefficient greater than 0.25. In this study, sample size was estimated according to the principle proposed by Kendall (24), which recommends a sample size of 5 to 10 times the number of variables. Because the core statistical analyses in this study involved 35 variables, the required sample size was estimated to be 175 to 350 participants. Given the longitudinal design of the study and an anticipated attrition rate of 20%, the minimum required sample size was 210. All final networks constructed in this study met the minimum stability criterion.

### 2.2 Instruments

#### 2.2.1 General information questionnaire

A self designed general information questionnaire was used to collect demographic and disease related data. Demographic variables included age, body mass index, marital status, place of residence, educational level, occupation, and medical payment method. Disease related variables included family history, smoking history, alcohol use history, previous transurethral resection of the prostate (TURP), presence of chronic diseases, clinical stage, Gleason score, and adjuvant therapy.

#### 2.2.2 The Chinese version of the memorial symptom assessment scale

The Memorial Symptom Assessment Scale is a multidimensional instrument originally developed by Portenoy et al. (25) in 1994 to assess symptoms in patients with cancer and other chronic illnesses. The Chinese version was translated by Cheng et al. (26) in Hong Kong and has demonstrated good reliability and validity, with Cronbach’s alpha coefficients ranging from 0.79 to 0.87. The scale consists of three parts. The first part includes 24 symptoms and assesses symptom occurrence (present or absent), frequency, severity, and distress. Symptom frequency is rated from rarely to almost constantly, severity from slight to very severe, and distress from not at all to very much. Frequency and severity are scored on a 1 to 4 scale, whereas distress is scored on a 0 to 4 scale. Higher scores indicate more frequent symptoms, greater severity, and greater distress. The second part includes 8 symptoms and assesses only symptom severity and distress, using the same scoring approach as in the first part. The third part contains supplementary items that allow respondents to report symptoms not listed in the main scale, and these items are scored in the same way as those in the first part. Based on a review of the literature, clinical practice, and discussions within the research team, three prostate cancer-specific symptoms, namely urinary incontinence, urinary frequency, and urinary urgency, were added to the survey to improve its suitability for assessing symptoms in radical prostatectomy patients. In the present study, the Cronbach’s alpha coefficients of the scale at T1, T2, and T3 were 0.812, 0.835, and 0.827, respectively.

### 2.3 Data collection

Data were collected using questionnaires. Before participant recruitment, all investigators received standardized training to ensure consistency in survey administration and instructions. Eligible participants were informed of the purpose and significance of the study, the questionnaire completion procedures, and relevant considerations. The voluntary nature of participation and the confidentiality of participants’ information were also emphasized. Questionnaires were administered only after written informed consent had been obtained. Participants completed the survey at three time points: 7 days after surgery (T1), 1 month after surgery (T2), and 3 months after surgery (T3).

### 2.4 Statistical analysis

#### 2.4.1 Descriptive analysis

Data were entered and analyzed using Excel and SPSS version 27.0. Means and standard deviations were used to describe demographic characteristics and symptom severity.

#### 2.4.2 Network analysis

##### 2.4.2.1 Network estimation

In this study, symptoms with a prevalence of at least 20% were included in the network analysis (27, 28). This threshold was applied to exclude infrequently reported symptoms with limited variability, which may produce unstable correlation estimates, while retaining symptoms that were sufficiently common to support reliable network estimation and improve network interpretability. The qgraph package in R was used to generate symptom networks based on the correlation matrix of symptoms, and the Fruchterman–Reingold force directed layout was applied for visualization, so that nodes with stronger connections were positioned closer together in the network graph (29). In the network, symptoms were represented as nodes, and the edges between nodes represented the correlations between symptoms. Edge thickness and color intensity reflected the strength of the associations between symptoms, with thicker and darker edges indicating stronger correlations.

##### 2.4.2.2 Network centrality indices

Centrality indices were calculated using the qgraph package, and centrality plots were generated using the centralityPlot function. The indices included strength, closeness, and betweenness centrality. Strength centrality was defined as the sum of the absolute weights of all edges connected to a given node. A higher strength value indicates that the symptom has stronger direct associations with other symptoms and occupies a more central position in the network (30). Strength centrality is considered the most important indicator for identifying core symptoms. When rankings differ across centrality measures, the ranking based on strength centrality is generally used as the primary criterion (31, 32). Therefore, in this study, core symptoms were identified on the basis of strength centrality.

##### 2.4.2.3 Network stability and accuracy

The bootnet package was used to assess network accuracy and stability. Centrality stability was evaluated using a case-dropping subset bootstrap procedure. In each iteration, a proportion of cases was randomly removed, and the correlation between the centrality estimates obtained from the subset and those obtained from the original sample was calculated. Stability was quantified using the correlation-stability (CS) coefficient. A CS coefficient greater than 0.25 was considered acceptable, whereas a value greater than 0.50 was considered good (32). Edge-weight accuracy was assessed using nonparametric bootstrapping. A total of 1, 000 bootstrap samples were drawn with replacement to estimate the 95% confidence intervals of the edge weights (32). Narrower confidence intervals indicated greater accuracy of the edge-weight estimates.

## 3 Results

### 3.1 General characteristics of radical prostatectomy patients

At T1, a total of 300 questionnaires were collected. Because of loss to follow up, voluntary withdrawal, and invalid data, 274 questionnaires were collected at T2 and 248 at T3. At baseline, 300 radical prostatectomy patients were included, all of whom were male. Most patients were aged 60 to 80 years (n = 251, 83.7%), married (n = 290, 96.7%), living in urban areas (n = 250, 83.3%), had a junior high school education (n = 91, 30.3%), were retired (n = 173, 57.7%), and were covered by employee health insurance (n = 216, 72.0%). In terms of body mass index, most patients had a BMI below 24 (n = 151, 50.3%).A family history of disease, smoking history, and alcohol use history were reported in 27 patients (9.0%), 137 patients (45.7%), and 115 patients (38.3%), respectively. In addition, 190 patients (63.3%) had comorbid chronic diseases, and 22 patients (7.3%) had a history of transurethral resection of the prostate. Regarding disease characteristics, clinical stage ≥ T2c was the most common (n = 107, 35.7%), and a Gleason score of 7 was the most frequently observed (n = 125, 41.7%). A total of 86 patients (28.7%) received adjuvant therapy. Detailed general characteristics are presented in **Table 1**.

**Table 1:** Characteristics of the participants (n=300).

Variables	Category	n (%)
Age	<60	34 (11.3)
	60–80	251(83.7)
	>80	15 (5.0)
BMI	<24	151(50.3)
	≥24	149(49.7)
Marital status	Unmarried	2 (0.7)
	Married	290(96.7)
	Widowed	7 (2.3)
	Divorced	1 (0.3)
Place of residence	Urban	250(83.3)
	Rural	50 (16.7)
Education level	Primary school or below	82 (27.3)
	Junior high school	91 (30.3)
	High school or technical secondary school	84 (28.0)
	College degree or above	43 (14.3)
Occupation	Employed	36 (12.0)
	Retired	173(57.7)
	Farmer	34 (11.3)
	Other	57 (19.0)
Medical payment method	Employee medical insurance	216(72.0)
	Resident medical insurance	84 (28.0)
Family history	No	273(91.0)
	Yes	27 (9.0)
Smoking history	No	163(54.3)
	Yes	137(45.7)
Alcohol use history	No	185(61.7)
	Yes	115(38.3)
Comorbid chronic disease	No	110(36.7)
	Yes	190(63.3)
History of TURP	No	278(92.7)
	Yes	22 (7.3)
Clinical stage	≤T2a	102(34.0)
	T2b	91 (30.3)
	≥T2c	107(35.7)
Gleason score	≤6	70 (23.3)
	7	125(41.7)
	8	60 (20.0)
	≥9	45 (15.0)
Adjuvant therapy	No	214(71.3)
	Yes	86 (28.7)

### 3.2 Symptom prevalence and severity at different time points in radical prostatectomy patients

At T1, a total of 12 symptoms had a prevalence of at least 20%. The three most severe symptoms were problems with sexual interest or activity (3.83 ± 0.40), pain (2.05 ± 0.90), and difficulty sleeping (1.75 ± 1.01). At T2, 10 symptoms had a prevalence of at least 20%, and the three most severe symptoms were problems with sexual interest or activity (2.54 ± 1.23), urinary incontinence (2.51 ± 1.30), and worrying (1.35 ± 1.07). At T3, 10 symptoms also had a prevalence of at least 20%, and the three most severe symptoms were problems with sexual interest or activity (2.07 ± 1.37), difficulty sleeping (1.42 ± 1.20), and urinary incontinence (1.40 ± 1.13). The prevalence and severity of symptoms at different time points are presented in **Table 2**.

**Table 2:** Prevalence and severity of symptoms at different time points among radical prostatectomy patients.

Symptom	T1 (n=300) n (%)	Severity (mean ± SD)	T2 (n=274) n (%)	Severity (mean ± SD)	T3 (n=248) n (%)	Severity (mean ± SD)
Difficulty concentrating	28 (9.33)	0.15 ± 0.49	7 (2.55)	0.04 ± 0.25	5 (2.02)	0.03 ± 0.23
Pain	278 (92.67)	2.05 ± 0.90	67 (24.45)	0.45 ± 0.83	36 (14.52)	0.26 ± 0.67
Lack of energy	221 (73.67)	1.27 ± 0.91	88 (32.12)	0.62 ± 0.95	89 (35.89)	0.66 ± 0.95
Cough	83 (27.67)	0.46 ± 0.80	50 (18.25)	0.31 ± 0.70	59 (23.79)	0.40 ± 0.76
Feeling nervous	64 (21.33)	0.27 ± 0.55	121 (44.16)	0.72 ± 0.88	57 (22.98)	0.40 ± 0.77
Dry mouth	171 (57.00)	0.97 ± 0.94	50 (18.25)	0.32 ± 0.87	51 (20.56)	0.31 ± 0.67
Nausea	19 (6.33)	0.10 ± 0.42	1 (0.36)	0.01 ± 0.12	1 (0.40)	0.01 ± 0.13
Feeling drowsy	12 (4.00)	0.06 ± 0.30	7 (2.55)	0.04 ± 0.28	1 (0.40)	0.00 ± 0.06
Numbness/tingling in hands/feet	3 (1.00)	0.02 ± 0.17	6 (2.19)	0.04 ± 0.27	6 (2.42)	0.04 ± 0.27
Difficulty sleeping	248 (82.67)	1.75 ± 1.01	158 (57.66)	1.28 ± 1.23	162 (65.32)	1.42 ± 1.20
Feeling bloated	128 (42.67)	0.85 ± 1.08	35 (12.77)	0.25 ± 0.67	18 (7.26)	0.14 ± 0.52
Vomiting	13 (4.33)	0.07 ± 0.35	0 (0.00)	0.00 ± 0.00	1 (0.40)	0.02 ± 0.25
Shortness of breath	80 (26.67)	0.42 ± 0.77	38 (13.87)	0.25 ± 0.67	37 (14.92)	0.27 ± 0.69
Diarrhoea	8 (2.67)	0.05 ± 0.33	3 (1.09)	0.03 ± 0.25	5 (2.02)	0.05 ± 0.37
Feeling sad	22 (7.33)	0.11 ± 0.41	32 (11.68)	0.16 ± 0.47	35 (14.11)	0.19 ± 0.51
Sweats	79 (26.33)	0.41 ± 0.75	44 (16.06)	0.27 ± 0.65	35 (14.11)	0.25 ± 0.66
Worrying	100 (33.33)	0.51 ± 0.82	189 (68.98)	1.35 ± 1.07	95 (38.31)	0.74 ± 1.04
Itching	0 (0.00)	0.00 ± 0.00	26 (9.49)	0.18 ± 0.58	25 (10.08)	0.18 ± 0.56
Lack of appetite	35 (11.67)	0.20 ± 0.59	29 (10.58)	0.20 ± 0.59	19 (7.66)	0.13 ± 0.48
Dizziness	60 (20.00)	0.33 ± 0.70	14 (5.11)	0.09 ± 0.39	22 (8.87)	0.17 ± 0.59
Difficulty swallowing	0 (0.00)	0.00 ± 0.00	0 (0.00)	0.00 ± 0.00	0 (0.00)	0.00 ± 0.00
Feeling irritable	2 (0.67)	0.01 ± 0.13	8 (2.92)	0.04 ± 0.25	5 (2.02)	0.02 ± 0.18
Problems with sexual interest or activity	300 (100.00)	3.83 ± 0.40	241 (87.96)	2.54 ± 1.23	187 (75.40)	2.07 ± 1.37
Problems with urination	0 (0.00)	0.00 ± 0.00	10 (3.65)	0.08 ± 0.44	13 (5.24)	0.10 ± 0.46
Mouth sores	2 (0.67)	0.01 ± 0.08	2 (0.73)	0.01 ± 0.09	4 (1.61)	0.02 ± 0.13
Change in the way food tastes	11 (3.67)	0.04 ± 0.19	8 (2.92)	0.03 ± 0.17	3 (1.21)	0.01 ± 0.11
Weight loss	13 (4.33)	0.05 ± 0.25	9 (3.28)	0.05 ± 0.32	10 (4.03)	0.07 ± 0.37
Hair loss	6 (2.00)	0.02 ± 0.14	1 (0.36)	0.00 ± 0.06	5 (2.02)	0.03 ± 0.23
Constipation	34 (11.33)	0.20 ± 0.60	61 (22.26)	0.43 ± 0.85	36 (14.52)	0.27 ± 0.69
Swelling of arms or legs	27 (9.00)	0.12 ± 0.40	2 (0.73)	0.02 ± 0.22	3 (1.21)	0.02 ± 0.21
“I don’t look like myself”	5 (1.67)	0.02 ± 0.13	0 (0.00)	0.00 ± 0.00	2 (0.81)	0.01 ± 0.09
Changes in skin	10 (3.33)	0.07 ± 0.67	7 (2.55)	0.03 ± 0.19	5 (2.02)	0.02 ± 0.18
Urinary incontinence	0 (0.00)	0.00 ± 0.00	235 (85.77)	2.51 ± 1.30	177 (71.37)	1.40 ± 1.13
Urinary frequency	0 (0.00)	0.00 ± 0.00	133 (48.54)	1.04 ± 1.17	67 (27.02)	0.54 ± 0.96
Urinary urgency	0 (0.00)	0.00 ± 0.00	83 (30.29)	0.67 ± 1.09	50 (20.16)	0.40 ± 0.85

### 3.3 Symptom networks at different time points in radical prostatectomy patients

**Figure 1:**
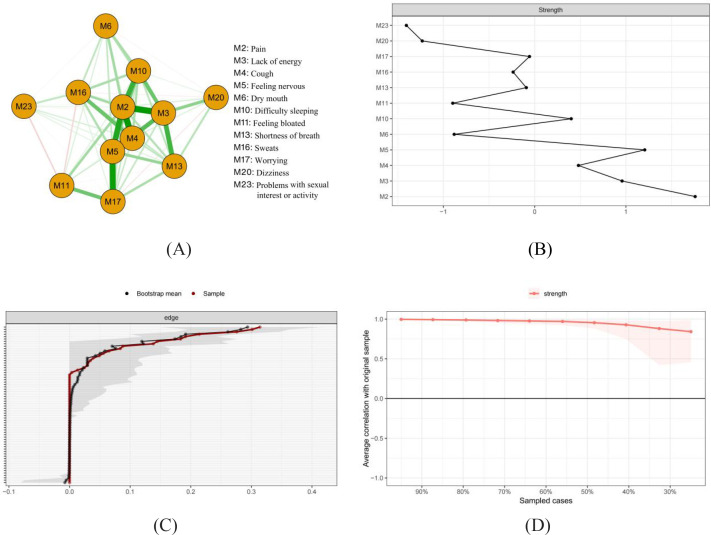
Symptom network and network robustness analysis at T1. **(A)** Symptom network. Nodes represent symptoms with a prevalence of at least 20%, and edges represent associations between symptoms; thicker edges indicate stronger associations. Green and red edges indicate positive and negative associations, respectively. **(B)** Strength centrality, with higher values indicating stronger overall connections with other symptoms. **(C)** Accuracy of edge-weight estimates based on nonparametric bootstrap confidence intervals. **(D)** Stability of strength centrality based on the case-dropping bootstrap procedure.

**Figure 2:**
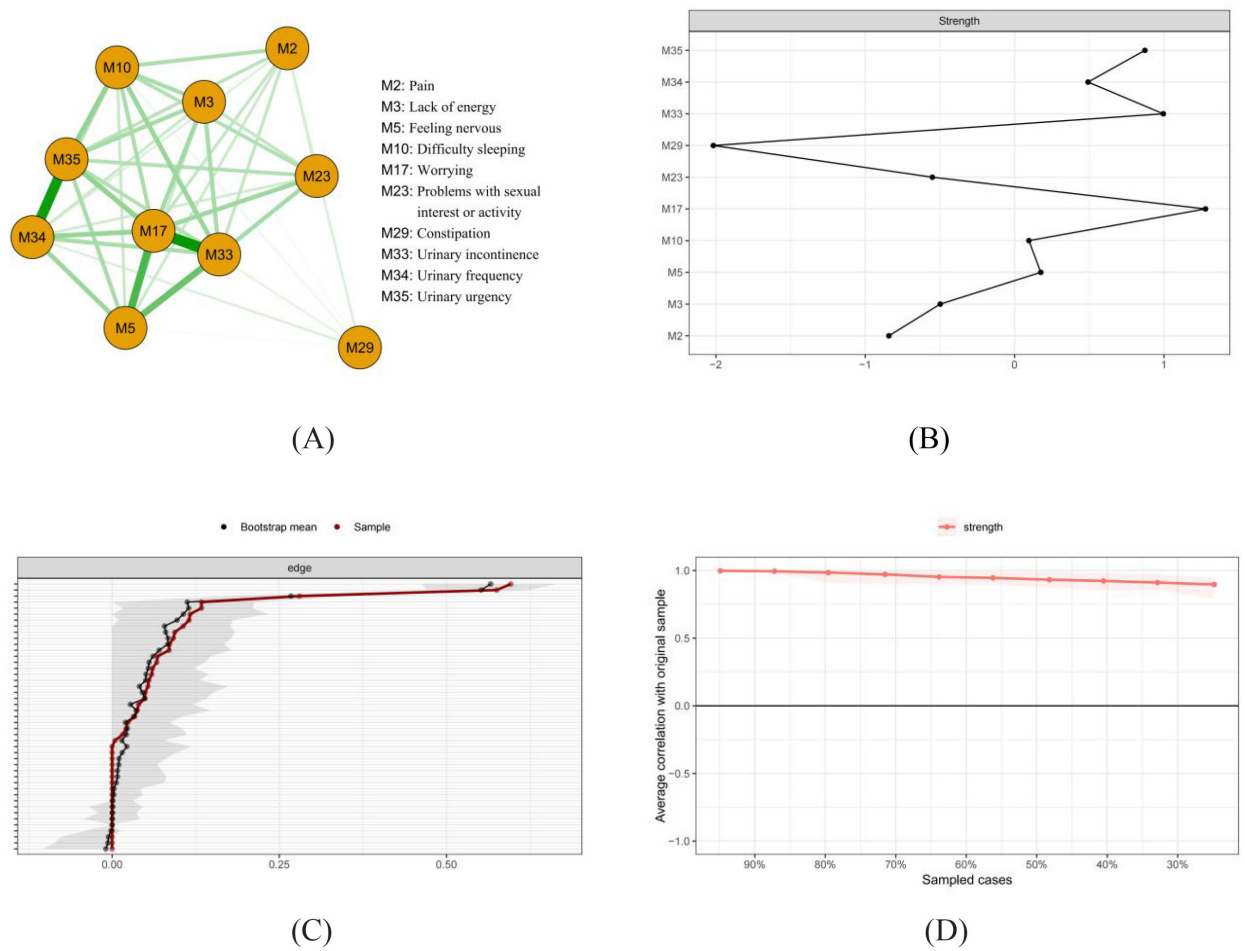
Symptom network and network robustness analysis at T2. **(A)** Symptom network. Nodes represent symptoms with a prevalence of at least 20%, and edges represent associations between symptoms; thicker edges indicate stronger associations. Green and red edges indicate positive and negative associations, respectively. **(B)** Strength centrality, with higher values indicating stronger overall connections with other symptoms. **(C)** Accuracy of edge-weight estimates based on nonparametric bootstrap confidence intervals. **(D)** Stability of strength centrality based on the case-dropping bootstrap procedure.

**Figure 3:**
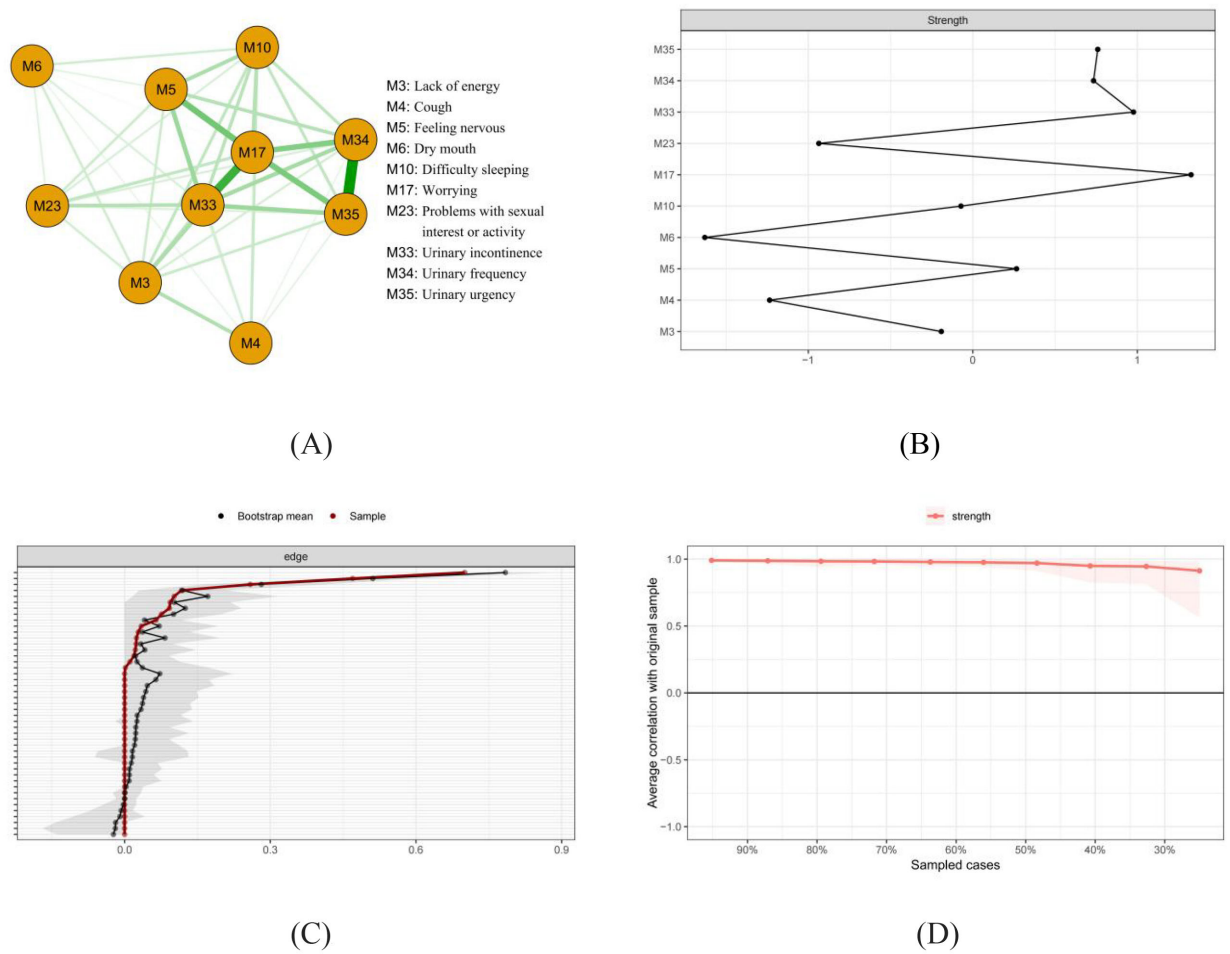
Symptom network and network robustness analysis at T3. **(A)** Symptom network. Nodes represent symptoms with a prevalence of at least 20%, and edges represent associations between symptoms; thicker edges indicate stronger associations. Green and red edges indicate positive and negative associations, respectively. **(B)** Strength centrality, with higher values indicating stronger overall connections with other symptoms. **(C)** Accuracy of edge-weight estimates based on nonparametric bootstrap confidence intervals. **(D)** Stability of strength centrality based on the case-dropping bootstrap procedure.

The symptom networks at T1, T2, and T3 are shown in **Figures 1**–**3**, respectively. The strongest symptom associations at T1, T2, and T3 were pain and lack of energy (M2–M3, r = 0.47), worrying and urinary incontinence (M17–M33, r = 0.72), and urinary frequency and urinary urgency (M34–M35, r = 0.88), respectively. At T1, the three symptoms with the highest strength centrality were pain (M2, rs = 1.76), feeling nervous (M5, rs = 1.21), and lack of energy (M3, rs = 0.96). At T2, the three symptoms with the highest strength centrality were worrying (M17, rs = 1.28), urinary incontinence (M33, rs = 1.00), and urinary urgency (M35, rs = 0.87). At T3, the three symptoms with the highest strength centrality were worrying (M17, rs = 1.32), urinary incontinence (M33, rs = 1.00), and urinary urgency (M35, rs = 0.74).

### 3.4 Stability and accuracy of symptom networks at different time points in radical prostatectomy patients

The results of the accuracy and stability analyses for the networks at T1, T2, and T3 are shown in **Figures 1**–**3**, respectively. The bootstrap confidence intervals at all time points were relatively narrow, indicating good network accuracy. The CS coefficients for T1, T2, and T3 were 0.517, 0.752, and 0.750, respectively, all of which were greater than 0.50, indicating that the constructed networks had good stability.

## 4 Discussion

### 4.1 Pain as the core symptom at 7 days after surgery in radical prostatectomy patients

Pain was identified as the core symptom at T1 in radical prostatectomy patients. At 7 days after surgery, patients are still in the recovery phase characterized by ongoing tissue repair and inflammatory responses. Incisional and deep tissue traction, localized edema, and sensitization of nerve endings make pain easily provoked or exacerbated during activities such as mobilization, turning in bed, and coughing. This activity related pain can directly interfere with patients’ ability to participate in early rehabilitation. Arnstein et al. (33) noted that postoperative movement related pain can substantially limit early ambulation and functional training, thereby delaying recovery and becoming an important barrier to postoperative functional restoration.

The network structure also showed relatively strong associations between pain and symptoms such as fatigue, cough, and sleep disturbance. Persistent pain can activate the sympathetic nervous system and the stress response axis, producing a state of heightened arousal and disrupting sleep continuity. This places the body in a high consumption, low recovery state (34), which can directly contribute to fatigue from a physiological perspective. At the same time, pain exerts a clear inhibitory effect on behavior. To avoid traction related pain, patients often reduce out of bed activity, walking, and rehabilitation exercises, resulting in reduced activity tolerance, diminished muscle strength, and poorer cardiopulmonary endurance, all of which further aggravate fatigue. In turn, fatigue weakens patients’ capacity to participate in activity and rehabilitation training, leading to slower recovery and more persistent pain related functional limitations. Postoperative patients are usually encouraged to perform deep breathing, effective coughing, and sputum clearance to maintain ventilation and facilitate secretion removal. However, pain may cause them to adopt shallow breathing and avoid forceful coughing, thereby creating a protective pattern of respiratory inhibition (35). When deep breathing is inadequate, coughing is weak, or cough behavior is suppressed, airway secretions are more likely to be retained, which increases airway irritation and may trigger more frequent or more severe coughing. Coughing itself can then place traction on the wound, increase intra abdominal pressure, and cause trunk vibration, thereby further provoking or worsening pain.

Therefore, pain should be regarded as a priority target for intervention in the early postoperative period. Timely and effective pain management can not only directly relieve suffering, but also alleviate related symptoms such as fatigue, cough, and sleep disturbance by reducing activity limitation and sleep disruption. Clinically, both resting pain and movement related pain should be assessed dynamically, with particular attention to pain during turning in bed, getting out of bed, deep breathing, and effective coughing, so that analgesic strategies can be adjusted in a timely manner. Pain management should follow multimodal and individualized principles, aiming to achieve optimal pain control with minimal adverse effects and to ensure that patients are able to participate effectively in physical activity. For patients with pronounced movement related pain, appropriate preemptive analgesia may be provided before activity to enable them to complete early rehabilitation within a tolerable range. At the same time, pain management should be integrated with rehabilitation nursing. When guiding patients in deep breathing, coughing and sputum clearance, and early ambulation, nursing staff should combine incision protection, positioning adjustments, and stepwise activity instruction to reduce the inhibitory effect of traction pain on activity. For patients who avoid activity because of pain, targeted bedside education should be strengthened, and activity goals should be broken down into manageable steps and advanced gradually. This approach may help improve ventilation and sputum clearance, reduce worsening fatigue and delayed recovery caused by insufficient activity, increase patients’ participation in early rehabilitation, and ultimately promote recovery in postoperative quality of life. To translate this finding into routine postoperative care, structured assessments of both resting and movement-evoked pain should be incorporated into follow-up during the first postoperative week to guide timely adjustment of analgesic strategies and rehabilitation support.

### 4.2 Worrying as the core symptom at 1 month and 3 months after surgery in radical prostatectomy patients

At both 1 and 3 months after surgery, worrying remained the core node in the symptom network, indicating that it occupied a central position during early postoperative recovery. At T2, the strongest association was observed between worrying and urinary incontinence, suggesting that uncertainty regarding the recovery of urinary control may be an important factor associated with patients’ worries during the early post-discharge period. At T3, although the strongest overall association in the symptom network was observed between urinary frequency and urinary urgency, worrying remained strongly connected with urinary incontinence and retained the highest strength centrality. It should be noted, however, that worrying was assessed using a general symptom item from the Memorial Symptom Assessment Scale rather than a urinary function-specific instrument, and the specific content or sources of patients’ worries were not further assessed. Therefore, the central position of worrying cannot be attributed exclusively to postoperative urinary dysfunction; it may also reflect uncertainty regarding sexual recovery, postoperative prostate-specific antigen surveillance, disease recurrence, and the overall recovery process.

Nevertheless, the close association between worrying and urinary incontinence suggests that urinary control may represent an important component of patients’ postoperative concerns. Urinary incontinence is often sudden, unpredictable, and recurrent. Patients may have difficulty anticipating when or under what circumstances urinary leakage will occur and may also be unable to clearly judge their progress in recovery. This perceived loss of control over bodily function may contribute to tension, uneasiness, and worry (36, 37). During daily activities, outings, and social interactions, patients may remain highly attentive to their urinary status, prepare protective products in advance, and worry about the inconvenience and embarrassment caused by urinary leakage. Such persistent vigilance and coping demands may further intensify psychological distress (38, 39). Postoperative sexual dysfunction may also affect intimate relationships, self-image, and perceptions of the male role, thereby adding to patients’ emotional burden (37).

Worrying may, in turn, be associated with greater attention to bodily changes and increased sensitivity to symptom fluctuations (40, 41). During the period from 1 to 3 months after surgery, some patients continue to experience urinary frequency, urinary urgency, sleep disturbance, urinary incontinence, and sexual dysfunction; sleep disturbance has also been reported to be associated with urinary symptoms following radical prostatectomy (42). Patients who remain concerned about their recovery may be more likely to interpret temporary discomfort or fluctuations in function as evidence of inadequate recovery or possible disease progression (43, 44). Such heightened vigilance may increase the perceived burden of symptoms, undermine confidence in recovery, and reduce patients’ ability to engage proactively in self-management. However, because the symptom networks were estimated separately at each time point, the temporal and causal directions of these associations cannot be determined. Worrying may represent a psychological response to persistent functional impairment, while also coexisting with and potentially reinforcing patients’ subjective experience of other symptoms.

The possible focus of worrying may also change across different stages of recovery, although this interpretation should be made cautiously because the specific content of worrying was not directly assessed. At T2, patients may be particularly concerned about managing early postoperative symptoms. During this period, urinary control remains unstable, and some patients may not yet have fully mastered self-management strategies such as pelvic floor muscle training, fluid management, and the use of protective products. These difficulties may contribute to uncertainty and a perceived lack of control. By T3, patients’ concerns may increasingly involve the pace and completeness of longer-term functional recovery. Previous studies have shown that urinary control may remain suboptimal in some patients at 3 months after radical prostatectomy (45, 46). When actual recovery does not meet preoperative expectations, patients may become concerned about persistent urinary dysfunction, future quality of life, sexual recovery, postoperative PSA results, or the possibility of disease recurrence.

These findings support the incorporation of stage-specific psychological assessment into routine postoperative follow-up. At 1 and 3 months after surgery, brief screening for anxiety and psychological distress should be accompanied by exploration of the specific sources of patients’ worries, including urinary control, sexual recovery, PSA surveillance, and fear of recurrence. Validated instruments such as the Memorial Anxiety Scale for Prostate Cancer or the Hospital Anxiety and Depression Scale may be used when clinically indicated. Based on the assessment results, healthcare professionals may provide individualized education, expectation management, pelvic floor muscle training, symptom self-management support, and relaxation strategies. Patients with persistent or clinically significant psychological distress should be referred for appropriate psychological or psychiatric evaluation.

This study has several limitations. First, all symptoms were self-reported, and worrying was assessed using a single general item from the Memorial Symptom Assessment Scale. Therefore, recall and reporting bias may have occurred, and the specific sources of patients’ worries could not be identified. Second, detailed surgical, pathological, and postoperative data, including nerve-sparing status, pelvic lymph-node dissection, catheterization duration, pathological stage, surgical margin status, postoperative PSA, and objective continence measures, were not prospectively and uniformly collected. Consequently, urinary symptoms reflected patient-reported symptom burden rather than objective urinary function, and subgroup differences according to surgical characteristics or postoperative outcomes could not be examined. Third, recruitment from two tertiary hospitals and attrition during follow-up may limit the generalizability of the findings. Finally, networks were estimated separately at each time point, precluding temporal or causal inference. Future multicenter prospective studies should integrate repeated patient-reported assessments with objective urinary measures, detailed clinical data, and longitudinal network methods.

## 5 Conclusion

This study used network analysis to examine changes in core symptoms at different time points during the early postoperative period in radical prostatectomy patients. The results showed that pain was the core symptom at T1, whereas worrying was the core symptom at both T2 and T3. Healthcare professionals should develop continuous, anticipatory, and targeted intervention strategies based on the core symptom identified at each time point, identify symptom risks as early as possible, and implement precision symptom management to improve patients’ symptom experience during early postoperative recovery and enhance the efficiency of symptom management.

## Data Availability

The raw data supporting the conclusions of this article will be made available by the authors, without undue reservation.
